# Supplementary material for: Classification Scheme of Heating Risk during MRI Scans on Patients with Orthopaedic Prostheses
Source: Diagnostics (Basel). 2022 Aug 2;12(8):1873. doi: 10.3390/diagnostics12081873 (PMC9406867; doi:10.3390/diagnostics12081873)
Supplement: Supplementary file 1 [file diagnostics-12-01873-s001.zip › diagnostics-1832548-supplementary.pdf]

# Classification Scheme of Heating Risk During MRI Scans on Patients with Orthopaedic Prostheses

# SUPPLEMENTARY MATERIALS

**Table S1**. Features of the Register of the Orthopaedic Prosthetic Implants (RIPO) database.

| Implant type | Data collection Time range | Included implants | Excluded implants | Number (approx.) |
|---|---|---|---|---|
| hip | 1st Jan. 2000 – 31st Dec. 2017 | total arthroplasty, hemiarthroplasty, resurfacing, revision and removal operations | special prostheses for tumour surgery | 175000 |
| knee | 1st Jan. 2000 – 31st Dec. 2017 | uni-, bi- and tri-compartimental arthroplasty, revision and removal operations | special prostheses for tumour surgery | 105000 |
| shoulder | 1st Jan. 2008 – 31st Dec. 2017 | anatomical and reverse arthroplasty, resurfacing, partial, revision and removal operations | special prostheses for tumour surgery | 6700 |

**Table S2.** Features of the Italian regional healthcare systems databases *Assistenza Specialistica Ambulatoriale* (ASA) and *Scheda di Dimissione Ospedaliera* (SDO). Abbreviation: SSN, *Sistema Sanitario Nazionale* (Italian National Healthcare System).

| **Clinical services database** | **Included patients services** | **Excluded patients services** |
|---|---|---|
| **ASA** | Collects specialist outpatient assistance provided to individual patients by public and private providers of the region accredited by the SSN | Specialist assistance given during hospital staying |
| **SDO** | Includes all the clinical services provided to individual patients during hospital staying by public and private hospitals of the region accredited by the SSN | |
